# Chemical Profiles and Protective Effect of *Hedyotis diffusa* Willd in Lipopolysaccharide-Induced Renal Inflammation Mice

**DOI:** 10.3390/ijms161126021

**Published:** 2015-11-13

**Authors:** Jian-Hong Ye, Meng-Hua Liu, Xu-Lin Zhang, Jing-Yu He

**Affiliations:** 1 Department of Endocrinology, Foshan Hospital of Traditional Chinese Medicine, Foshan 528000, China; yejianhong2005@163.com; 2 Guangdong Provincial Key Laboratory of New Drug Screening, School of Pharmaceutical Sciences, Southern Medical University, Guangzhou 510515, China; 3 State Key Laboratory of Organ Failure Research, Guangdong Provincial Institute of Nephrology, Southern Medical University, Guangzhou 510515, China; 4 Bioengineering Research Center, Guangzhou Institute of Advanced Technology, Chinese Academy of Sciences, Guangzhou 511458, China; zxl_lxz@live.com

**Keywords:** *Hedyotis diffusa* Willd, chemical profiles, renal inflammation, flavonoids, iridoids, anthraquinones

## Abstract

Protective effect of *Hedyotis diffusa* (*H. diffusa*) Willd against lipopolysaccharide (LPS)-induced renal inflammation was evaluated by the productions of cytokines and chemokine, and the bioactive constituents of *H. diffusa* were detected by the ultra-fast liquid chromatography -diode array detector-quadrupole-time of flight mass spectrometry (UFLC-DAD-Q-TOF-MS/MS) method. As the results showed, water extract of *H. diffusa* (equal to 5.0 g/kg body weight) obviously protected renal tissues, significantly suppressed the productions of tumor necrosis factor-α (TNF-α), interleukin (IL)-1β, IL-6, and monocyte chemoattractant protein (MCP)-1, as well as significantly promoted the production of IL-10 in serum and renal tissues. According the chemical profiles of *H. diffusa*, flavonoids, iridoid glycosides and anthraquinones were greatly detected in serum from *H. diffusa* extract treatment mice. Two main chemotypes, including eight flavonoids and four iridoid glycosides were found in renal tissues from *H. diffusa* extract treatment mice. The results demonstrated that water extract of *H. diffusa* had protective effect on renal inflammation, which possibly resulted from the bioactive constituents consisting of flavonoids, iridoids and anthraquinones.

## 1. Introduction

*Hedyotis diffusa* (*H. diffusa*) Willd, an annual slender plant belong to *Rubiaceae*, is widely distributed in South of China, and other Asian country, such as Indonesia, Japan, Malaysia, Nepal, Philippines, Sri Lanka and Thailand [1]. *H. diffusa* named “*Bai Hua She She Cao*” in Chinese, is a famous traditional Chinese Medicine and applied on treating bronchitis, arthritis, rheumatism, appendicitis, sore throat, urethral infection, contusions, ulcerations and extension of malignancies [2]. Pharmacological studies show that *H. diffusa* has anticancer, anti-inflammatory, antioxidative, neuroprotective, hepatoprotective, anti-mutagenesis, and immunemodulating activities [2,3,4,5,6]. Meanwhile, phytochemical studies have shown that the major constituents of *H. diffusa* are anthraquinones, flavonoids and iridoid glycosides [7].

As known, the chemical constituents are responsible for the efficacy of herbal medicine. There are some investigations focused on traditional use of *H. diffusa* extract, the investigation on evaluating the anti-inflammatory effect and searching the anti-inflammatory constituents, especially for renal inflammation, have been rarely found during the past two decades. Clarifying which constituents absorbed into blood to produce anti-inflammatory effect plays a key role in clinical use of *H. diffusa*. However, the bioactive constituents are generally minor or trace after absorbed into blood. To elucidate the anti-inflammatory constituents from *H. diffusa*, the method that could improve resolution efficiency and enhance the signal response of minor/trace constituents should be used.

Ultra-fast liquid chromatography-diode array detector-quadrupole-time of flight mass spectrometry (UFLC-DAD-Q-TOF-MS/MS) is a very useful means for separation and identification of compounds from herbal extract and biological samples. The UFLC-DAD-Q-TOF-MS/MS method has been so far used to analyze the constituents in *H. diffusa* due to the high resolution and low detection limit [8,9]. This method, however, has not been used for research on anti-inflammatory constituents of *H. diffusa* *in vivo*.

In the study, the protective effect of *H. diffusa* on anti-inflammation was evaluated using histological appearance and immunohistochemistry of renal sections from lipopolysaccharide (LPS)-induced renal inflammation mice model. The levels of pro-inflammatory cytokines, anti-inflammatory cytokine and chemokine in serum and renal tissues were detected to evaluate the anti-inflammatory effect of *H. diffusa*. Furthermore, to find out the potential bioactive constituents with anti-inflammatory effect, serum and renal chemical profiles of *H. diffusa* were studied by UFLC-DAD-Q-TOF-MS/MS method.

## 2. Results and Discussion

### 2.1. Effect of Water Extract of H. diffusa on the Histology of LPS-Induced Renal Inflammation Mice

**Figure 1:**
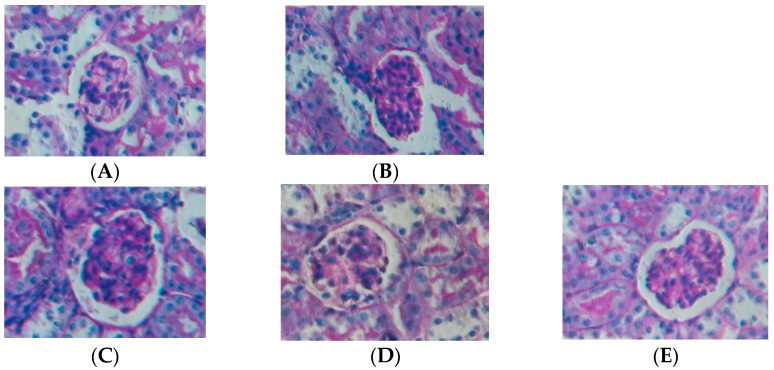
Effect of *Hedyotis diffusa* extract in the LPS-treated mice: histological appearance of renal sections of mice treated with saline (**A**); LPS (**B**); low dose (1.25 g/kg body weight) (**C**); medium dose (2.5 g/kg bw) (**D**); and high dose (5.0 g/kg bw) (**E**) of water extract of *H. diffusa*. The renal sections were analyzed by PAS staining (magnification is 400×).

No histological changes were seen in renal section of the control group (Figure 1). In contrast, histological evaluation of renal sections from LPS-treated group revealed that necrotic epithelial cells, invasion of inflammatory cells in the interstitium and swelling glomeruli with decrease of capsular space. Comparing to LPS-treated group, the administration of low and medium doses of water extract of *H. diffusa* (1.25 and 2.5 g/kg body weight (bw)) partially prevented renal damage induced by LPS. The high dose (5.0 g/kg bw) could have better protection to the mice renal tissue from damage induced by LPS.

**Figure 2:**
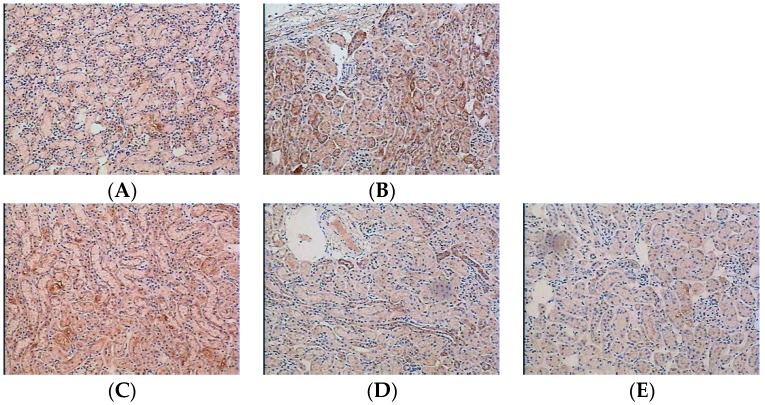
Effects of *H. diffusa* extract on the infiltration of macrophage in the kidneys: CD68 positive cells stained with immunohistochemistry are shown after treatment with (**A**) saline; (**B**) LPS; (**C**) low dose, 1.25 g/kg bw; (**D**) medium dose, 2.5 g/kg bw; and (**E**) high dose, 5.0 g/kg bw) (magnification is 200×).

Macrophages involvement in LPS-induced renal damage was assessed, and therefore the glycoprotein CD68, one of important antigens for macrophage study, was examined after LPS-treated mice. As shown in Figure 2, CD68-positive macrophages were at low levels in control group. Compared with the after LPS-treated group, water extract of *H. diffusa* obviously reduced the CD68, indicating that the number of infiltrative macrophage was markedly inhibited after *H. diffusa* treatment.

### 2.2. Effect of Water Extract of H. diffusa on the Productions of Cytokines and Chemokines

**Figure 3:**
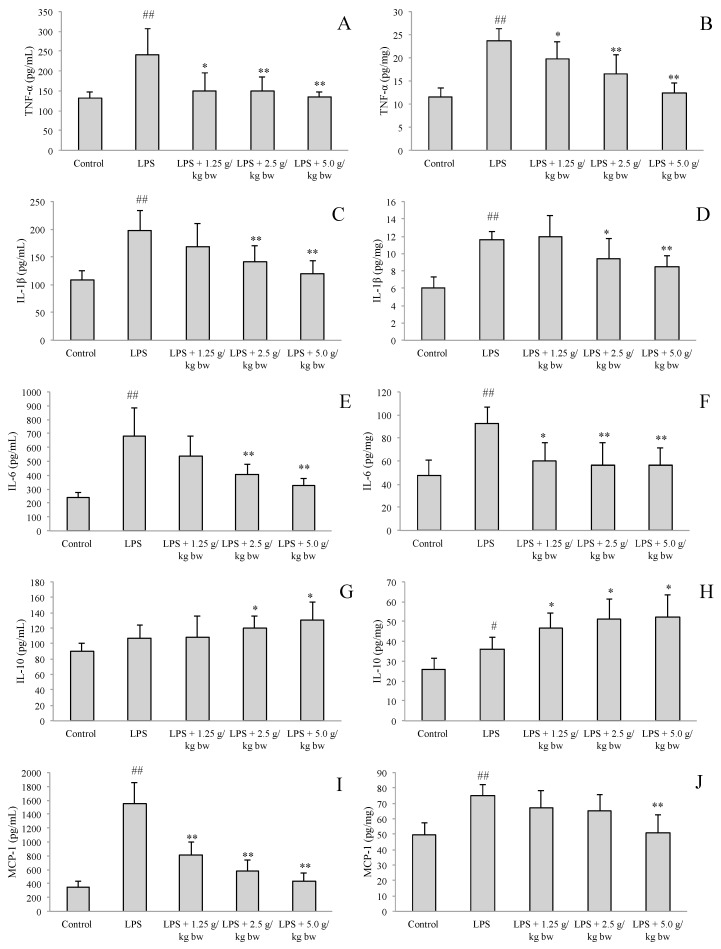
Effects of water extract of *H. diffusa* on the levels of TNF-α (**A**,**B**), IL-1β (**C**,**D**), IL-6 (**E**,**F**), IL-10 (**G**,**H**), and MCP-1 (**I**,**J**) in serum and renal tissue of mice. Results are expressed as means ± SD (*n* = 8). * *p* < 0.05, ** *p*< 0.01 compared with LPS group; ^#^ *p* < 0.05, ^##^ *p* < 0.01 compared with control group.

To elucidate the protective effect of *H. diffusa* on LPS-induced renal inflammation, the levels of important cytokines tumor necrosis factor-α (TNF-α), interleukin (IL)-1β, IL-6, and chemokine monocyte chemoattractant protein (MCP)-1 in serum and renal tissues were measured (Figure 3). Injection of LPS caused a significant increase in the levels of pro-inflammatory cytokines TNF-α, IL-1β, IL-6, and MCP-1 in serum and renal tissues and the level of anti-inflammatory cytokine IL-10 in renal tissues as compared with those in the control group. The administration of low dose of water extract of *H. diffusa* (1.25 g/kg bw) could decrease in the level of TNF-α in serum and renal tissues, and partially affect the factors in serum or renal tissues, including significant increase in the level of IL-10, decrease in the level of IL-6 in renal tissues and the level of MCP-1 in serum compared with that of the LPS-treated group. The medium dose treated group (2.5 g/kg bw) could significantly decrease the level of TNF-α, IL-1β and IL-6, and increase the level of IL-10 in serum and renal tissues; moreover, this dose (2.5 g/kg bw) could also significantly decrease the level of MCP-1 in serum when compared with that of the LPS-treated group. Treatment with high dose of *H. diffusa* (5.0 g/kg bw) could significantly decrease productions of TNF-α, IL-1β, IL-6, and MCP-1, and increased production of IL-10 as compared with those in the LPS-treated group, showing the dose-dependent manner. In addition, the high dose treated group showed no significant difference to the control group in the levels of TNF-α, IL-1β, IL-6, and MCP-1 in serum and renal tissues, while this group had significantly higher level of IL-10 than that of the control group.

### 2.3. Chemical Profiles of Water Extract of H. diffusa by UFLC-DAD-Q-TOF-MS/MS

**Figure 4:**
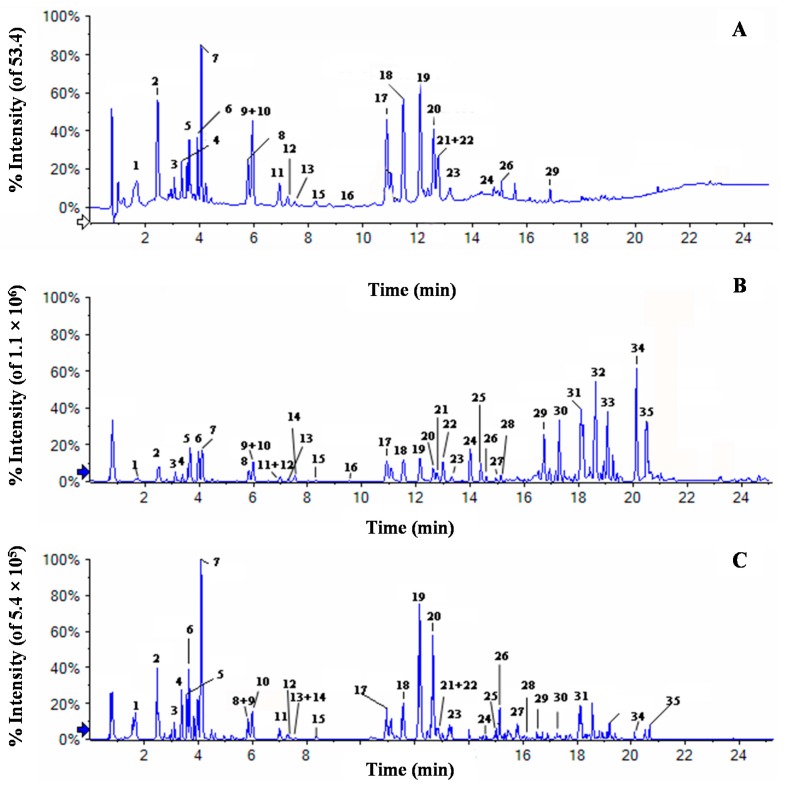
Chromatograms of water extract of *H. diffusa*: (**A**) DAD chromatogram detected at 254 nm; (**B**) total ion chromatogram in positive ion mode; and (**C**) total ion chromatogram in negative ion mode). White arrow means DAD chromatogram (**A**) and the blue arrows means MS chromatogram in positive and negative ion mode (**B**,**C**).

**Figure 5:**
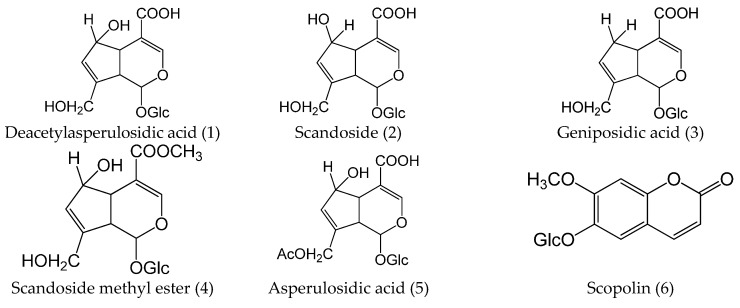

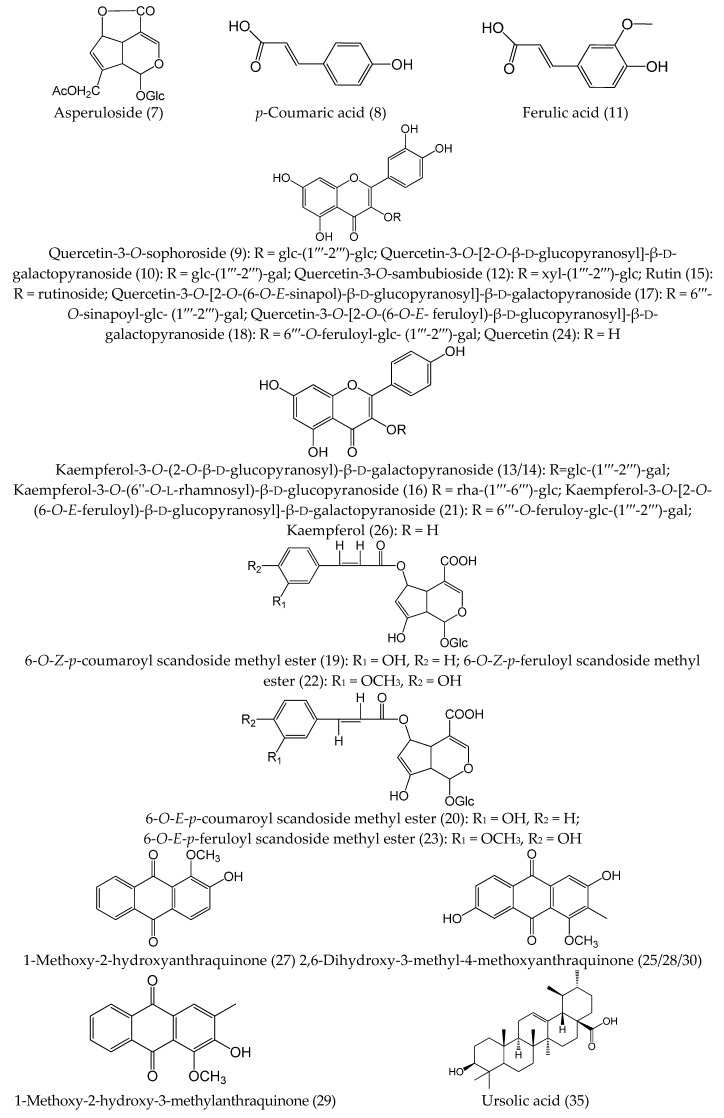
Chemical structures of the compounds identified in *H. diffusa* extract, mice plasma and kidney.

In this study, water extract of *H. diffusa* was detected both in positive ion mode and negative ion mode (Figure 4). The detailed information of 35 compounds has been listed in Table 1 and their chemical structures were shown in Figure 5. Simple peaks 1, 2, 3, 4, 5, 6, 7, 8, 11, 15, 24, 26 and 35 have unequivocally identified as deacetylasperulosidec acid, scandoside, geniposidic acid, scandoside methyl ester, asperulosidic acid, scopolin, asperuloside, *p*-courmaric acid, ferulic acid, rutin, quercetin, kaemperol and ursolic acid, respectively, by comparing their retention time, UV data, molecular weight and MS spectra with those of standards. The constituents that were with no referable standards have been tentatively characterized with UV data, molecular weight, and fragment behaviors in MS spectra and from the literature.

Due to abundant capillary in mice abdomen, constituents in *H. diffusa* could be absorbed quickly and comprehensively by intraperitoneal injection. After deducting the matrix interference from control plasma and model plasma, most of these compounds, except peaks 6, 22, 23, 33 and 35, were also found in dosed plasma. However, only peaks 1, 2, 3, 4, 9, 10, 13, 14, 16, 18, 24, 26 and 27 were detected in kidney homogenate by XIC manager (Extract Ions Using Dialog). Peaks 31, 32, and 34 were found in bio-samples from control and model group as well as from dosed group. In view of the fragmentor rule, they were inferred as polyunsaturated fats compounds. The tentative identification of each components were outlined below.

6-*O*-*Z*-*p*-Coumaroyl scandoside methyl ester (peak 19) and 6-*O*-*E*-*p*-coumaroyl scandoside methyl ester (peak 20) were isomers with [M − H]^−^ ions at *m*/*z* 549.16274 and *m*/*z* 549.16396 (C_26_H_30_O_13_), respectively, in negative ion mode. Peaks 22 and 23 were identified as 6-*O*-*Z*-*p*-feruloyl scandoside methyl ester and 6-*O*-*E*-*p*-feruloyl scandoside methyl ester, 30 Da more than peak 19. The produced fragment ion at *m*/*z* 223, *m*/*z* 193, and *m*/*z* 119 from these four compounds were similar to scandoside methyl ester. It indicated that the four compounds were the derivatives of scandoside methyl ester, which is in accordance with previous literature [8,9].

Flavonoids and its derivatives showed characteristic UV spectra and fragment rules in mass spectra under collision induced decomposition condition [10,11]. The groups linked to aglycone, such as glucose, galactose and rhamnose, were always loss firstly and the deprotonated aglycone always had a high relative aboundance. Compounds 9 and 10 were a pair of isomers with the protonated molecular ions at *m*/*z* 627.15557 and *m*/*z* 627.15525 (C_27_H_30_O_17_), respectively, and similar fragmentations, showing the occurrence of two hexosyl residue. Referring to the literature [12], they were tentatively identified as quercetin-3-*O*-sophoroside and quercetin-3-*O*-[2-*O*-β-d-glucopyranosyl]-β-d-galactopyranoside. Compound 12 gave protonated ion at *m*/*z* 597.14508 (C_26_H_28_O_16_). The MS/MS analyses showed the loss of 132 Da and 294 Da (132 + 162), suggesting the presence of xylose and glucose groups. Therefore, it was identified as quercetin-3-*O*-sambubioside in according with previous report [13]. Compound 17 was characterized as quercetin-3-*O*-[2-*O*-(6-*O*-*E*-sinapol)-β-d-glucopyranosyl]-β-d-galactopyranoside with deprotonated molecular ion at *m/z* 831.19906 (C_38_H_40_O_21_), 206 Da higher than that of compound 10, suggesting that it was a sinapol derivated of 10 [9,13]. In addition, the base peak at *m*/*z* 301.0344 demonstrated the presence of quercetin. Compound 18 gave deprodonated molecular ion at *m*/*z* 801.18894 (C_37_H_38_O_20_), with similar MS/MS spectra with compound 17. It was 30 Da less than that of compound 17, indicating the lack of a methoxyl group. Thus, it was tentatively characterized as quercetin-3-*O*-[2-*O*-(6-*O*-*E*-feruloyl)-β-d-glucopyranosyl]-β-d-galactopyranoside [12]. Compounds 13, 14, 16 and 21 showed the characteristic MS fragmentation of kaempferol derivated. The loss of 324 Da (162 + 162) for 13 and 14, 306 Da (146 + 162) for 16 and 500 Da (176 + 162 + 162) indicated different groups linked to kaempferol aglycone. Because of the lack of more evidence, compounds 13 and 14 were tentatively characterized as Kaempferol-3-*O*-(2-*O*-β-d-glucopyranosyl)-β-d-galactopyranoside and its isomer [12,14], and need to be confirmed by NMR. Compounds 16 and 21 were tentatively characterized as kaempferol-3-*O*-(6′′-*O*-l-rhamnosyl)-β-d-glucopyranoside and kaempferol-3-*O*-[2-*O*-(6-*O*-E-feruloyl)-β-d-glucopyranosyl]-β-d-galactopyranoside, respectively [12,15].

**Table 1:** Chemical profiles in *H diffusa* by ultra-fast liquid chromatography-diode array detector-quadrupole-time of flight mass spectrometry.

No.	Rt (min)	Molecular Formula	λmax (nm)	[M + Na]^+^	[M + H]^+^	[M – H]^−^	Major Fragmentors in Positive Mode	Major Fragmentors in Negative Mode	Identification	Source
1	1.62	C_16_H_22_O_11_	239	413.10505 (−0.9)		389.10862(−0.8)	395.0879 [M+Na–H_2_O]^+^, 251.0512 [M+Na–glc]^+^, 233.0420 [M+H–glc]^+^, 215.0299 [M+H–glc–H_2_O]^+^	227.0579 [M–H–glc]^−^, 209.0468 [M–H–glc–H_2_O]^−^, 183.0683 [M–H–glc–CO_2_]^−^, 165.0573 [M–H–glc–CO_2_–H_2_O]^−^, 137.0616 [M–H–glc–CO_2_–H_2_O–CO]^−^	Deacetylasperulosidic acid	Herb, Plasma, Kidney
2	2.50	C_16_H_22_O_11_	238	413.10505 (−0.9)		389.10862 (−0.8)	395.0948 [M+Na–H_2_O]^+^, 233.0408 [M+H–glc]^+^	227.0588 [M–H–glc]^−^, 209.0473 [M–H–glc–H_2_O]^−^, 183.0679 [M–H–glc–CO_2_]^−^, 165.0573 [M–H–glc–CO_2_–H_2_O]^−^, 147.0464 [M–H–glc–CO_2_–2H_2_O]^−^, 139.0411 [M–H–glc–2CO_2_]^−^, 121.0302 [M–H–glc–2CO_2_–H_2_O]^−^, 89.0253, 59.0165	Scandoside	Herb, Plasma, Kidney
3	3.01	C_16_H_22_O_10_	235	397.11053 (+0)		373.11388 (−0.4)	235.0561 [M+Na–glc]^+^, 217.0439, 191.0673	211.0620 [M–H–glc]^−^, 167.0707 [M–H–glc–CO_2_]^−^, 149.0606, 123.0453	Geniposidic acid	Herb, Plasma, Kidney
4	3.38	C_17_H_24_O_11_	238	427.12095 (−0.3)		403.12433 (−0.6)	265.0669 [M+Na–glc]^+^	241.0710 [M–H–glc]^−^, 223.0628 [M–H–glc–H_2_O]^−^, 209.0444 [M–H–glc–CH_3_OH]^−^, 193.0683, 191.0508, 167.0350, 139.0397	Scandoside methyl ester	Herb, Plasma, Kidney
5	3.60	C_18_H_24_O_12_	229	455.11562 (−0.8)		431.11967 (+0.4)	437.1027 [M+Na–H_2_O]^+^, 293.0621 [M+Na–glc]^+^, 275.0518 [M+H–glc–H_2_O]^+^, 197.0191, 147.0430	269.0669 [M–H–glc]^−^, 251.0588 [M–H–glc–H_2_O]^−^, 225.0792 [M–H–glc–CO_2_]^−^, 179.0566 [M–H–glc–CO_2_–HCOOH]^−^, 165.0573 [M–H–glc–CO_2_–CH_3_COOH]^−^, 121.0302 [M–H–glc–2CO_2_–CH_3_COOH]^−^, 89.0257, 59.0170	Asperulosidic acid	Herb, Plasma
6	3.98	C_16_H_18_O_9_	221, 320		355.10270 (+1.0)	353.0881 (+0.8)	163.0384, 145.0238, 135.0442, 117.0343, 89.0398	191.0552 [M–H–glc]^−^, 179.0341, 135.0446	Scopolin	Herb
7	4.13	C_18_H_22_O_11_	238	437.10509 (−0.3)		413.1085 (−0.9)	275.0542 [M+Na–glc]^+^, 187.0372, 147.0458	251.0544 [M–H–glc]^−^, 205.0529 [M–H–glc–HCOOH]^−^, 191.0364 [M–H–glc–CH_3_COOH]^−^, 147.0457 [M–H–glc–CH_3_COOH–CO_2_]^−^, 119.0501 [M–H–glc–CH_3_COOH–CO–CO_2_]^−^	Asperuloside	Herb, Plasma
8	5.79	C_9_H_8_O_3_	219, 296		165.05416 (−2.8)	163.04128 (7.4)	147.0435 [M+H–H_2_O]^+^, 119.049 [M+H–HCOOH]^+^, 91.0552, 77.0406, 65.0415	119.0505 [M–H–CO_2_]^−^, 93.0355	*p*-Coumaric acid	Herb, Plasma
9	5.85	C_27_H_30_O_17_	256, 355	649.1370 (−0.8)	627.15557 (+)	625.14120 (+0.3)	465.1052 [M+H–glc]^+^, 303.0490 [M+H–2glc]^+^	301.0383 [M–H–2glc]^−^, 271.0277 [M–H–2glc–CH_2_O]^−^	Quercetin-3-*O*-sophoroside	Herb, Plasma, Kidney
10	5.99	C_27_H_30_O_17_	256, 355	649.1370 (-0.8)	627.15525 (−0.5)		465.1021 [M+H–gal]^+^, 303.0493 [M+H–gal–glc]^+^	301.0317 [M–H–gal–glc]^−^, 271.0280 [M–H–gal–glc–HCOH]^−^	Quercetin-3-*O*-[2-*O*-β-d-glucopyranosyl]-β-d-galactopyranoside	Herb, Plasma, Kidney
11	6.92	C_10_H_10_O_4_	219		195.06477 (−2.1)	193.05146 (+4.3)	177.0549 [M+H–H_2_O]^+^, 149.0523 [M+H–HCOOH]^+^ 145.0283, 89.0396	149.0457 [M–H–CO_2_]^−^	Ferulic acid	Herb, Plasma
12	7.01	C_26_H_28_O_16_	264, 339	619.12649 (-0.8)	597.14508 (+0.1)	595.13022 (−0.4)	465.1011 [M+H–xyl]^+^, 303.0484 [M+H–xyl–glc]^+^	301.0341 [M–H–xyl–glc]^−^, 271.0246 [M–H–xyl–glc–CH_2_O]^−^	Quercetin-3-*O*-sambubioside	Herb, Plasma
13	7.27	C_27_H_30_O_16_	265, 344	633.14211 (−0.8)	611.16075 (+0.1)	609.14959 (+5.6)	449.1062 [M+H–glc]^+^, 287.0540 [M+H–glc–gal]^+^, 163.0598	285.0348 [M–H–glc–gal]^−^, 255.0320 [M–H–glc–gal–CH_2_O]^−^	Kaempferol-3-*O*-(2-*O*-β-d-glucopyranosyl)-β-d-galactopyranoside or isomer	Herb, Plasma, Kidney
14	7.57	C_27_H_30_O_16_	264, 340	633.14211 (−0.8)	611.16069 (0)	609.15134 (+8.6)	449.1081 [M+H–glc]^+^, 287.0541 [M+H–glc–gal]^+^, 145.0482	285.0434 [M–H–glc–gal]^−^, 255.0320 [M–H–glc–gal–CH_2_O]^−^	Kaempferol-3-*O*-(2-*O*-β-d-glucopyranosyl)-β-d-galactopyranoside or isomer	Herb, Plasma, Kidney
15	8.31	C_27_H_30_O_16_	265, 340	633.14211 (−0.8)	611.16069 (0)	609.15128 (+8.5)	465.1016 [M+H–rha]^+^, 303.0489 [M+H–rha–glc]^+^	301.0377 [M–H–rha–glc]^−^	Rutin	Herb, Plasma
16	9.39	C_27_H_30_O_15_	265, 340		595.16572 (0)	593.15110 (-0.2)	449.1026 [M+H–rha]^+^, 287.0548 [M+H–rha–glc]^+^	285.0459 [M–H–rha–glc]^−^	Kaempferol-3-*O*-(6′′-*O*-L-rhamnosyl)-β-d-glucopyranoside	Herb, Plasma, Kidney
17	10.92	C_38_H_40_O_21_	265, 338	855.19472 (−0.8)	833.21331 (−0.2)	831.19906 (+0.2)	465.1032303.0499 [M+H–sinapol–glc]^+^, 303.0499 [M+H–sinapol–glc-gal]^+^, 177.0489	625.1414 [M–H–sinapol]^−^, 301.0344 [M–H–sinapol–glc–gal]^−^, 271.0250	Quercetin-3-*O*-[2-*O*-(6-*O*-*E*-sinapol)-β-d-glucopyranosyl]-β-d-galactopyranoside	Herb, Plasma
18	11.54	C_37_H_38_O_20_	254, 336	825.18454 (−0.4)	803.20329 (+0.2)	801.18894 (+0.7)	303.0493 [M+H–feruloyl–glc-gal]^+^, 177.0539	625.1477 [M–H–feruloyl]^−^, 301.0391 [M–H–feruloyl–glc–gal]^−^, 271.0285	Quercetin-3-*O*-[2-*O*-(6-*O*-*E*-feruloyl)-β-d-glucopyranosyl]-β-d-galactopyranoside	Herb, Plasma, Kidney
19	12.16	C_26_H_30_O_13_	309	573.15785 (+0)	551.17641 (+0.9)	549.16274 (+2.5)		369.1011 [M–H–glc–H_2_O]^−^, 223.0634 [M–H–glc–H_2_O–cou]^−^, 193.0552, 191.0369, 163.0409, 119.0519	6-*O*-*Z*-*p*-coumaroyl scandoside methyl ester	Herb, Plasma
20	12.64	C_26_H_30_O_13_	309	573.15785 (+0)	551.17589 (0)	549.16396 (+4.7)	225.0723 [M+H–glc–H_2_O–cou]^+^	369.0978 [M–H–glc–H_2_O]^−^, 223.0616 [M–H–glc–H_2_O–cou]^−^, 193.0547, 191.0361, 163.0403, 119.0510	6-*O*-*E*-*p*-coumaroyl scandoside methyl ester	Herb, Plasma
21	12.78	C_37_H_38_O_19_	268, 328	809.18960 (−0.4)	787.20863 (+0.8)	785.19407 (+0.8)	449.1072 [M+H–fer–glc]^+^, 287.0553 [M+H–fer–glc–gal]^+^, 177.0542, 145.0275	609.1492 [M–H–fer]^−^, 429.0849 [M–H–fer–glc–H_2_O]^−^, 285.0431 [M–H–fer–glc–gal]^−^, 255.0303, 227.0367	Kaempferol-3-*O*-[2-*O*-(6-*O*-*E*-feruloyl)-β-d-glucopyranosyl]-β-d-galactopyranoside	Herb, Plasma
22	12.87	C_27_H_32_O_14_	299	603.16862 (+0.3)	581.1864 (−0.2)	579.17408 (3.7)		399.1111 [M–H–glc–H_2_O]^−^, 223.0617 [M–H–fer–glc–H_2_O]^−^, 193.0532, 191.0377, 134.0391, 119.0350	6-*O*-*Z*-*p*-feruloyl scandoside methyl ester	Herb
23	13.26	C_27_H_32_O_14_	299	603.16862 (+0.3)	581.18627 (−0.4)	579.17283 (+1.5)		399.1083 [M–H–glc–H_2_O]^−^, 223.0622 [M–H–fer–glc–H_2_O]^−^, 193.0518, 191.0375, 134.0382, 119.0348	6-*O*-*E*-*p*-feruloyl scandoside methyl ester	Herb
24	14.64	C_15_H_10_O_7_	254, 370		303.04973 (−0.7)	301.03565 (+0.9)	285.0385 [M+H–H_2_O]^+^, 229.0479 [M+H–H_2_O–2CO]^+^, 177.0543, 153.0176	286.0583 [M–H–CH_3_]^−^, 179.0001, 151.0058	Quercetin	Herb, Plasma, Kidney
25	14.78	C_16_H_12_O_5_			285.07565 (−0.3)	283.06143 (+0.8)	267.0651 [M+H–H_2_O]^+^, 239.0722 [M+H–H_2_O–CO]^+^, 209.0580 [M+H–H_2_O–CO–HCOH]^+^, 181.0650 [M+H–H_2_O–2CO–HCOH]^+^, 153.0697	268.0399 [M–H–CH_3_]^−^ , 239.0364 [M–H–CO_2_]^−^, 211.0432 [M–H–CO_2_–CO]^−^, 195.0471 [M–H–2CO_2_]^−^	2,6-Dihydroxy-3-methyl-4-methoxyanthraquinone or isomer	Herb, Plasma
26	15.01	C_15_H_10_O_6_	260, 370		287.0548	285.04049 (+0.1)	165.0170, 153.0170, 121.0274	179.0001 [M–H–2CO_2_]^−^, 151.0025, 107.0129, 121.0284	Kaempferol	Herb, Plasma, Kidney
27	15.98	C_15_H_10_O_4_			255.06504 (−0.6)	253.05088 (+1.0)	227.0675 [M+H–CO]^+^, 209.0570 [M+H–CO–H_2_O]^+^, 199.0738 [M+H–2CO]^+^, 171.0792 [M+H–3CO]^+^, 153.0678 [M+H–3CO–H_2_O]^+^	224.0497 [M+H–CHO]^−^, 183.0670 [M+H–CHO–CH_3_CO]^−^	1-Methoxy-2-hydroxyanthraquinone	Herb, Plasma, Kidney
28	16.17	C_16_H_12_O_5_			285.07548 (−1.0)	283.05952 (−5.9)	270.0544 [M+H–CH_3_]^+^, 252.0400 [M+H–CH_3_–H_2_O]^+^, 224.0463 [M+H–CH_3_–H_2_O–CO]^+^ 196.0500 [M+H–CH_3_–H_2_O–2CO]^+^, 168.0568, 139.0521	268.0376 [M–H–CH_3_]^−^, 253.0146 [M+H–HCOH]^−^, 240.0432 [M–H–CH_3_–CO]^−^, 225.0202 [M+H–HCOH–CO]^−^, 212.0451 [M–H–CH_3_–2CO]^−^, 197.0311 [M+H–HCOH–2CO]^−^, 184.0539, 169.0316	2,6-Dihydroxy-3-methyl-4-methoxyanthraquinone or isomer	Herb, Plasma
29	16.93	C_16_H_12_O_4_			269.08036 (−1.8)	267.06684 (+2.1)	254.0562 [M+H–CH_3_]^+^, 226.0610 [M+H–CH_3_–CO]^+^, 197.0582 [M+H–CH_3_–CO–CHO]^+^, 181.0636, 152.0609	252.0534 [M+H–CH_3_]^−^, 224.0485 [M+H–CH_3_–CO]^−^	1-Methoxy-2-hydroxy-3-methylanthraquinone	Herb, Plasma
30	17.53	C_16_H_12_O_5_			285.0754	283.06143 (+0.8)	270.0521 [M+H–CH_3_]^+^, 242.0551 [M+H–CH_3_–CO]^+^, 214.0610 [M+H–CH_3_–2CO]^+^ 187.0594, 169.0646	268.0376 [M–H–CH_3_]^−^, 240.0432 [M–H–CH_3_–CO]^−^, 212.0451 [M–H–CH_3_–2CO]^−^	2,6-Dihydroxy-3-methyl-4-methoxyanthraquinone or isomer	Herb, Plasma
31	18.21	C_18_H_28_O_2_			277.21599 (−0.8)	275.20167 (+0.1)	259.2041, 235.1693, 171.1151, 149.1311, 135.1159, 121.1088, 107.0859, 93.0709, 79.0561	231.2161 [M–H–CO_2_]^−^	Unidentified	Herb, Plasma, Kidney
32	18.56	C_18_H_30_O_2_			297.23200 (+0.5)	277.21874 (+5.2)	261.2217, 243.2109, 223.1691, 187.1478, 173.1321, 151.1482, 137.1328, 123.1172, 109.1021, 95.0807, 81.0720		Unidentified	Herb, Plasma, Kidney
33	19.07	C_19_H_26_O_5_		357.16079 (−0.4)	335.18563 (+1.0)		163.0751, 145.0643, 115.0543, 91.0555, 71.0515		Unidentified	Herb
34	20.13	C_18_H_30_O_2_			279.23202 (+0.6)	277.21874 (+5.2)	261.2214, 243.2105, 223.1696, 187.1479, 173.1322, 151.1470, 137.1321, 123.1173, 109.1020, 95.0867, 81.0716, 67.0572	259.2064 [M–H–H_2_O]^−^, 233.2282 [M–H–CO_2_]^−^	Unidentified	Herb, Plasma, Kidney
35	20.68	C_30_H_48_O_3_			457.36734 (−0.6)	455.35279 (−0.6)	439.3564, 411.3615, 393.3508, 341.2846, 315.2694, 297.2575, 249.1844, 231.2106, 203.1791, 163.1476, 149.1316, 121.1009, 95.0863, 81.0713	407.3300 [M–H–HCOOH]^−^	Ursolic acid	Herb

As shown in Figure 3, anthraquinone compounds did not showed obviously UV absorption in DAD spectra, but with good mass response in positive ion mode. They were always characterized by losses of CH_3_ (15 Da) CO (28 Da), H_2_O (18 Da), and HCOH (30 Da). Compounds 25, 28 and 30 gave the same protonated molecular ion at *m/z* 285 in positive ion mode and *m*/*z* 283 in negative ion mode, with molecular formula of C_16_H_12_O_5_. The losses of neutral ions of 18 Da, 28 Da and 30 Da in MS spectra showed anthraquinone compounds characteristics. Thus they were identified as 2,6-Dihydroxy-3-methyl-4-methoxyanthraquinone or its isomer referring to previous report [16], and were necessary to be confirmed by NMR. Compound 27 gave protonated ion at *m*/*z* 255.06504 (C_15_H_10_O_4_), and 30 Da less than compound 25, suggesting the lack of methoxy group. Compound 29 showed molecular formula of C_16_H_12_O_4_ with the protonated ion at 269.08036, and 14 Da more than compound 27 suggested the presence of methyl group. In according with the report [17,18], compounds 27 and 29 were identified as 1-methoxy-2-hydroxyanthraquinone and 1-methoxy-2-hydroxy-3-methylanthraquinone, respectively.

### 2.4. Discussion

LPS as an important inflammatory factor, could induce endotoxemia, shock and multiple organ dysfunction syndromes. During LPS-induced inflammatory process, multiple endogenous inflammatory mediators were produced in response to LPS, and the immune responses and organic functions were modulated [19,20,21]. The inflammatory model induced by LPS was commonly used to investigate the effect of herbal medicines and its bioactive constituents on renal inflammation [21,22,23,24]. In various acute and chronic renal inflammations, renal tubular epithelial cells actively participate in the process of glomerular sclerosis and renal fibrosis via producing all kinds of inflammatory cytokines, chemokines and extracellular matrix [25]. It has been reported that macrophages are involved in the productions of pro-inflammatory cytokines [26]. Macrophage infiltration is considered to be a hallmark of all kinds of injury, and macrophage infiltration into the kidney is found as one of the initial events of renal disease [27]. In this study, the results of histological and *immunohistochemical* examination of renal section clearly showed the evidence that *H. diffusa* extract had protective effect against renal inflammation induced by LPS, at the dose-dependent manner.

TNF-α, as the pro-inflammatory cytokine, activates other cytokines and therefore plays an important role in the pathogenesis of LPS-induced renal damage. A way to protect against LPS-induced inflammation is to inhibit TNF-α action [28,29]. Similarly, IL-1β could also stimulate the production of other pro-inflammatory cytokines, such as IL-6 [30,31]. It is thought that increasing production of IL-1β in renal tissues indicates the pathological development of inflammation, while increasing production of IL-6 is involved in the chronic processes and can suppress the synthesis of IL-1β in the second phase of the inflammatory response [32]. *H. diffusa* could down-regulate the levels of pro-inflammatory cytokines in serum and renal tissue, therefore markedly ameliorating the local immune response induced by LPS. Moreover, TNF-α stimulates the production of chemokines, such as MCP-1. MCP-1 is chemotactic for multiple leukocytes, including neutrophils, monocytes and natural killer cells [33,34]. The anti-inflammatory cytokine IL-10, which suppresses the production of pro-inflammatory cytokines and chemokines mainly through inhibition of innate immune cell activation, was up-regulated and therefore ameliorated LPS-induced renal damage in animal models [35]. The inflammatory reaction was evaluated by the production levels of pro-inflammatory cytokines, anti-inflammatory cytokine and chemokine in serum and renal tissues. The results demonstrated that water extract of *H. diffusa* could dose-dependently attenuate the levels of TNF-α, IL-1β, IL-6, MCP-1, and IL-10, which are associated with the improvement of function in nephropathy tissues.

A series of studies showed that flavonoids, iridoids and anthraquinones from herbal medicine have anti-inflammatory effect [36,37,38,39,40,41]. Kaempferol could suppress the activation of inflammatory nuclear factor-κB (NF-κB) transcription factor through nuclear factor-inducing kinase/I κB kinase and mitogen-activated protein kinase (MAPK) in aged rat kidney [42]. Quercetin had the ability to attenuate activation of NF-κB and inhibited IL-1-triggered MCP-1 expression via suppression of NF-κB [43]. Rutin, a quercetin glycoside, attenuated renal inflammation and apoptosis by reducing NF-κB, TNF-α and caspase-3 expression [44]. The 5-glucoside of quercetin named saxifragin exerted anti-inflammatory activity by inhibiting NF-κB, caspase-1, and MAPK activation [45]. Emodin, an anthraquinone derivative could activate peroxisome proliferator-activated receptor γ (PPAR-γ), and inhibit TLR2-mediated NF-κB signal pathway, thereby contributing to the immune inflammation regulation of emodin in LPS-induced acute kidney injury [46,47]. Iridoid glycosides possessed anti-inflammatory and immunomodulatory effects by suppressing transmembrane signal transmission in NF-κ Bp65 pathway, decreasing the expressions of MCP-1 and α-smooth muscle actin (α-SMA) to modulate the productions of pro-inflammatory mediators in nephropathy tissue [48,49,50]. The studies showed that inhibition of inflammatory response and induction of apoptotic signal via natural products could be useful approaches for prevention and cure of LPS-induced renal damage. In this study, the chemical profiles indicated that water extract of *H. diffusa* mainly contained these three chemotypes by UPLC-DAD-Q-TOF-MS/MS method. As high doses of extract of *H. diffusa* obviously protected renal tissues, suppressed the productions of the anti-inflammatory cytokines and chemokine, and promoted the production of anti-inflammatory cytokine, chemical profiles of the absorbed constituents of *H. diffusa* were necessary to elucidate the effective compounds *in vivo*. In the study, there were ten iridois, eleven flavonoids and five anthraquinones found in serum from the *H. diffusa* extract treatment groups. Moreover, the flavonoids, including kaempferol, quercetin, three kaempferol glycosides and three quercetin glycosides, were the most chemotype found in nephropathy tissues, indicating that flavonoids might target on the nephropathy tissues and greatly contribute to the suppression of inflammation process. Four iridois and one anthraquinone found in renal tissues suggested that these constituents might also have anti-inflammatory effect. The results of chemical study indicated that flavonoids, iridoids and anthraquinones might be responsible for the anti-inflammatory effect of *H. diffusa* and therefore could ameliorate the renal function and regulate the levels of the cytokines and chemokines through anti-inflammatory responses. However, the quantification of potential bioactive constituents and the method validation are not performed due to lacks of two-third of commercial standards, including three iridois, nine flavonoids, five anthraquinones and three unidentified compounds. Isolation and structural identification of non-commercial potential bioactive constituents from *H. diffusa* are undergoing in order to exactly identify and completely quantify all potential bioactive constituents in serum and renal tissues. In addition, the molecular pathway and anti-inflammatory mechanism of *H. diffusa* extract are still unclear, and thus further study is required according to the previous reports and these results.

## 3. Experimental Section

### 3.1. Plant Material

The materials of *H. diffusa* were collected from Guangzhou Qingping Herbal Market, Guangzhou, China. The materials were authenticated by the authors and dried at room temperature. The materials were stored in Guangzhou Institute of Advanced Technology, Chinese Academy of Sciences, Guangzhou, China.

### 3.2. Chemicals and Reagents

Deacetylasperulosidec acid, scandoside, geniposidic acid, scandoside methyl ester, asperulosidic acid, scopolin and asperuloside were purchased from Chengdu Biopurify Technology Development Co., Ltd. (Chengdu, China). *p*-courmaric acid, ferulic acid, rutin, quercetin, kaempferol, ursolic acid were from National Institutes for food and drug control (Beijing, China). HPLC-grade methanol was purchased from Fisher Scientific (Pittsburgh, PA, USA). Distilled water was purified by a Milli-Q system (Millipore, Milford, MA, USA). Other reagents were analytical grade.

### 3.3. Sample Preparation

Thirteen standards for the identification were prepared in HPLC-grade methanol to obtain the standard solutions of deacetylasperulosidec acid (0.17 μg/mL), scandoside (0.21 μg/mL), geniposidic acid (0.20 μg/mL), scandoside methyl ester (0.19 μg/mL), asperulosidic acid (0.20 μg/mL), scopolin (0.18 μg/mL), asperuloside (0.20 μg/mL), *p*-courmaric acid (0.20 μg/mL), ferulic acid (0.19 μg/mL), rutin (0.21 μg/mL), quercetin (0.21 μg/mL), kaempferol (0.20 μg/mL), and ursolic acid (0.19 μg/mL), respectively. All standard solutions were stored at 4 °C until used.

The dried *H. diffusa* were cut into 1 cm pieces, weighed (100 g) and boiled in 1 L water for 1 h; after filtration, the extraction was repeated twice; the combined filtrates were evaporated in vacuum at 70 °C (EYELA N-1001, Tokyo, Japan). The concentrated solution (100 mL) was added to ethanol (300 mL) and stored at 4 °C overnight; after filtration, ethanol in the solution was evaporated at 50 °C; and, finally, 100 mL of a brown, concentrated extract (equal to 1.0 g raw material/mL) were obtained and stored at 4 °C until used.

### 3.4. Animals and Experiment Design

Kunming mice in the body weights of 18–20 g were purchased from the Experimental Animal Center of Southern Medical University (Guangzhou, China). Mice were fed on standard laboratory diet and water at *libitum*, and kept in 12 h dark/light cycle room at 21 ± 5 °C with a relative humidity of 55% ± 10% for one week before used. All experimental protocols were approved by the Animal Care and Use Committee of Southern Medical University (No. 2014A03031028, Guangzhou, China).

Mice were randomly divided into 5 groups (*n* = 8). In a week, the control group and the model group was injected intraperitoneally with sterile physiological saline (0.2 mL/20g bw, i.p. (intraperitoneal injection)); the treatment groups received the water extract of *H. diffusa* (equal to 1.25, 2.5 or 5.0 g/kg bw, i.p.). Except for the control group, the other four groups were injected intraperitoneally with LPS (3 mg/kg bw) after the sample injection on the fifth day. LPS injection was continued for three consecutive days. On the seventh day, all mice from each group were anesthetized by ether after LPS injection. Blood samples were taken from abdominal aorta. The serum was obtained by centrifugation at 13,000× *g* at 4 °C for 10 min and stored at −80 °C until analysis. After kidneys were removed carefully, one kidney was fixed with 10% formaldehyde for histological and immunohistochemical examinations, and the other one was frozen in liquid nitrogen for subsequent analysis. The frozen kidney was homogenized in ice-cold Tris–HCl buffer (pH = 7.4) containing protease inhibitors. The homogenized samples were centrifuged at 13,000× *g* at 4 °C for 20 min. The supernatants were collected and frozen at −80 °C until assay.

### 3.5. Preparation of Bioanalytical Samples

An aliquot of 160 μL of combined plasma (20 μL per mouse) was mixed with 600 μL acetonitrile and vortexed for 1 min. After centrifuging at 13,000× *g* at 4 °C for 30 min, the supernatant was transferred into a fresh tube and evaporated with nitrogen. The residue dissolved in a 160 μL mobile phase was vortexed for 3 min and centrifuged at 13,000× *g* for 30 min. Finally, an aliquot of 10 μL of the supernatant were injected into the UFLC/DAD/Q-TOF-MS/MS system. An aliquot of 160 μL of mixed kidney supernatants (20 μL per mouse) was prepared as same method as the combined plasma.

### 3.6. Bioanalytical Method

UFLC analysis was performed on a Shimadzu UFLC XR instrument (Shimadzu Corp., Kyoto, Japan), consisting of a binary pump, an autosampler, a column oven and a diode-array detector. Samples were separated on a Phenomenex Kinetex column (2.1 mm i.d. (internal diameter) × 100 mm, 2.6 μm, Phenomenex, CA, USA). The mobile phase consisted of water (A) and methanol (B) both containing 0.1% formic acid (*v*/*v*) using a gradient elution program of 13% B (0–2 min), 13%–15% B (2–8 min), 15%–23% B (8–12 min), 23%–98% B (12–20 min) and 98% B (20–25 min). A 2-min post-run time was set to equilibrate the column. The flow rate was kept at 0.3 mL/min. The injected volume was 2 μL and the column temperature was set at 40 °C. The DAD detector scanned from 190 to 400 nm. Mass spectrometry was performed on the Triple TOF™ 5600 plus (AB SCIEX, Foster City, CA, USA) a hybrid triple quadrupole time-of-flight mass spectrometer equipped with ESI source. The system was operated with Analyst^®^ TF 1.6 software (AB SCIEX, Foster City, CA, USA). The conditions of MS/MS detector were as follows: ion source gas 155 psi; ion source gas 255 psi; curtain gas 30 psi; Source temperature 550 °C; ion spray voltage floating 4500 V; collision energy 35 V; collision energy spread 15 V; declustering potential 80 V. TOF-MS range was set at *m*/*z* 100–1000 and product ions mass range was set at *m*/*z* 50–1000. Both positive and negative ion modes were used for compounds ionization. Nitrogen was used as nebulizer and auxiliary gas.

The UFLC-DAD-Q-TOF-MS/MS data of samples were extracted and analyzed by PeakView software (AB SCIEX, Foster City, CA, USA), mainly with the XIC manager tool that provided the quasi-molecular weight, mass errors and isotope pattern fit. The predicted formula with errors less than ± 5 ppm was applied to compare with that of the compounds reported in Rubiaceae. The tentative identification of each compound was further guided by MS/MS spectrum (AB SCIEX, Foster City, CA, USA) that could determine the possible elemental compositions of the fragment ions and propose the fragmentation pathways.

### 3.7. Histological Examination

Kidneys were fixed with 10% formalin for 24 h in a shaker and embedded in paraffin. The paraffin blocks were cut into 4-μm-thick sections using a microtome and then stained with periodic acid-Schiff (PAS) reagents for histological examination. Renal sections were evaluated at 400× magnification.

### 3.8. Immunohistochemical Examination

After embedded renal tissues were deparaffinised in xylene and rehydrated, and the immunohistochemical examination was performed according to the manufacturer’s instruction (GTVisionTMI Detection System, Gene Tech, Shanghai, China). Eight random fields were chosen and evaluated at 200× magnifications.

### 3.9. Determination of Cytokines

Concentrations of TNF-α, IL-1β, IL-6, IL-10 and MCP-1 in serum and renal tissues were determined using mouse-specific enzyme-linked immunosorbent assay (ELISA) kits (NeoBioscience, Shenzhen, China). Each analysis was performed according to the manufacturer’s instruction, and the concentrations of cytokines were calculated according to the standard curves.

### 3.10. Statistical Analysis

Results were shown as mean ± standard deviation (SD) for each experiment. The data obtained were analyzed using one-way analysis of variance (ANOVA) followed by Tukey’s multiple comparison test (SPSS version 19.0, IBM, New York, NY, USA). A *p* value <0.05 was considered as significant difference.

## 4. Conclusions

In this study, the results showed that water extract of *H. diffusa* could protect against the renal damage induced by LPS through down-regulating the levels of TNF-α, IL-1β, IL-6, and MCP-1, as well as up-regulating the level of anti-inflammatory cytokine IL-10. It also demonstrated that the main constituents, flavonoids, iridoids and anthraquinones, were possibly responsible for the anti-inflammatory effect of *H. diffusa*.
